# Incidence, patterns and management of complications (90-day-analysis) after liver surgery – a single center experience with 3177 consecutive hepatic resections

**DOI:** 10.1007/s00423-026-04077-4

**Published:** 2026-05-25

**Authors:** Verena Tripke, Fabian Bartsch, Arndt Weinmann, Tobias Huber, Eva-Verena Griemert, Michael B. Pitton, Janine Baumgart, Jens Mittler, Hauke Lang

**Affiliations:** 1 Department of General, Visceral and Transplantation Surgery, University Medical Center of the Johannes Gutenberg University Mainz, Langenbeckstraße 1, 55131 Mainz, Germany; 2 Department of General and Visceral Surgery, HospitaìBarmherzige Brüder`Brüder`Trier, Nordallee 1, 54292 Trier, Germany; 3 Department of Internal Medicine I, University Medical Center of the Johannes Gutenberg University Mainz, Langenbeckstraße 1, 55131 Mainz, Germany; 4 Department of Anesthesiology, University Medical Center of the Johannes Gutenberg University Mainz, Langenbeckstraße 1, 55131 Mainz, Germany; 5 Department of Diagnostic and Interventional Radiology, University Medical Center of the Johannes Gutenberg University Mainz, Langenbeckstraße 1, 55131 Mainz, Germany

**Keywords:** Liver surgery, Liver resection, Hepatectomy, Perioperative outcome, Liver tumor

## Abstract

**Purpose:**

Despite recent advances in surgical techniques and perioperative management, liver surgery is still associated with perioperative morbidity and mortality. This study aims to investigate the spectrum, risk factors and management of postoperative complications.

**Methods:**

All patients who underwent liver resection between 01/2008 until 12/2023 were identified from a prospective institutional database. The data was analyzed regarding postoperative morbidity and mortality rates.

**Results:**

We identified 3 177 liver resections including 1 345 (42.3%) major hepatectomies (≥ 3 segments). Complications occurred in 1 530 (48.2%). Of these, 460 (14.5%) were grade IIIa, 133 (4.2%) grade IIIb, 118 (3.7%) grade IVa, 13 grade IVb and 111 (3.5%) grade V. A total of 1 158 reinterventions were performed including 256 reoperations, 61 s operative revisions, 109 endoscopic procedures and 732 percutaneous interventions. In multivariate analysis age (*p* < 0.001), need for hepaticojejunostomy (*p* < 0.001), duration of operation (*p* = 0.001), and intraoperative blood transfusion (*p* < 0.001) were independent risk factors for mortality. Operative revision was associated with an increased mortality rate (26.6% vs. 1.5%, *p* < 0.001).

**Conclusion:**

The need for operative revision marked a subgroup with particularly poor outcome, emphasizing the importance of early detection, minimally invasive complication management whenever feasible, and careful risk stratification in complex liver surgery. Operative revision should be reserved for cases that cannot be managed successfully by intervention or when inevitable.

## Introduction

Liver surgery has become the standard of care for many primary liver malignancies as well as selected secondary liver malignancies, in particular colorectal liver metastases. In the 1970s, hepatic surgery was associated with mortality rates up to 20% [1]. Ongoing technical and medical innovations, a better understanding of hepatic anatomy, improved anesthesiologic management, careful patient selection, more specialization of hepatobiliary surgery in high-volume centers and progress in complication management have resulted in a continuous improvement of results of liver surgery. Despite these advances, the perioperative morbidity still approaches up to 50%, and perioperative mortality in general is still in the 2–6% range [2–6]. Population-based studies reveal an actually higher mortality rate than studies from high-volume hospitals. However, the comparability of results is often limited by a substantial heterogeneity of patient cohorts, a lack of or inconsistent use of definitions for morbidity periods (i.e. 30-day-mortality, 90-day-mortality, in hospital-mortality) [7]. Although there are several standard definitions of complications and their complexity such as the definition of posthepatectomy liver failure [8] and biliary leakage [9] or the Clavien-Dindo classification [10], their use has been very variable, which hinders comparability.

This study aims to analyze spectrum and risk factors for 90-day-perioperative morbidity and mortality after liver resection in a German high-volume liver center using standardized definitions. In addition, special attention was paid to the management of postoperative complications, especially interventional management.

## Material and methods

All cases of liver resection between January 2008 and December 2023 were identified using a prospective institutional database. Preoperative diagnostics usually include abdominal computed tomography (CT) or magnetic resonance imaging (MRI) and thoracic CT scans in cases of malignant disease. Furthermore, in selected cases with anticipated complex liver resection a three-dimensional reconstruction with liver volumetry or segmentation and even a full-sized 3D-print of the liver was performed for resection planning on request of the surgeon [11]. Endoscopic retrograde cholangiopancreatography (ERCP), percutaneous transhepatic cholangiography (PTC) or magnetic resonance cholangiopancreatography (MRCP) were performed in selected cases.

### Surgical technique

For open surgery, parenchymal dissection was performed by scratching the parenchyma with the scissor tip, by the clamp crushing technique or by using an ultrasound dissector. Small vessels were sealed with bipolar forceps and larger vessels with clips and sutures, whereas the hilar structures and hepatic veins were transected and sutured over vascular clamps. Parenchymal dissection in laparoscopic liver surgery was performed using an ultrasonic dissector or a laparoscopic Cavitron ultrasonic surgical aspirator. Major vascular structures, particularly the hilar plate or hepatic veins, were transected using endo-GIA staplers.

Intraoperative ultrasound was routinely performed, and the Pringle maneuver or total vascular occlusion was applied on special “request” of the surgeon. An abdominal drainage was usually placed.

Patients` characteristics, including age, gender, BMI (body-mass-index), ASA (American Society of Anesthesiologists) classification, cirrhosis/Child-Pugh score, comorbidities, intraoperative data (extent of liver resection, additional procedures, requirement of blood transfusion, estimated intraoperative blood loss), and postoperative outcomes were analyzed. In patients with cirrhosis, the Child-Pugh Score was calculated. Liver function capacity was assessed using the LiMAx test in selected cases only [12]. Major hepatectomy was defined as resection of ≥ 3 liver segments [5], and extended hepatectomy as resection of five or more segments.

Postoperative surgical complications were graded according to the Clavien-Dindo classification system [10]. Biliary leakage was classified using the ISGLS definition [9]. Posthepatectomy liver failure (PHLF) was assessed according to the ISGLS definition [8]. Additionally, posthepatectomy liver failure was assessed using the “50–50 criteria” (prothrombin time < 50% and serum bilirubin > 50 µmol/L on postoperative day 5; [13]) and using the definition of Mullen et al. (peak serum bilirubin > 7 mg/dl at any time; [14]). All complications that occurred during the initial perioperative hospital stay and the 90 day-readmission rates were recorded. Mortality was defined as perioperative 90-day mortality.

The analysis was stratified to three different time groups: Early era 2008–2012, mid era 2013–2018 and late era 2019–2023.

### Statistics

Categorical variables are presented as numbers and percentages, whereas continuous variables are expressed as median/range or mean/standard deviation (SD). Continuous variables were compared using the Mann–Whitney U test, and categorical variables were compared via the χ^2^-test. Binary logistic regression was used for multivariable analysis. Statistical significance was set at p-values < 0.05. SPSS Version 23 (IBM Corporation, USA) was used for statistical analysis.

## Results

A total of 3 177 liver resections to treat benign or malignant hepatobiliary diseases were performed from January 2008 to December 2023. Of these, 354 (11.1%) were repeated liver resections, 87 (2.7%) re-repeated, 32 four-time, 7 five-time resections and one six-time resection. A total of 345 (10.9%) resections were performed minimally-invasively. Liver cirrhosis was present in 194 (6.1%) patients, with 188 (97%) having Child A cirrhosis. Moreover, 985 (31%) of the cases had received chemotherapy/and or targeted therapy or lately immunotherapy prior to resection. Transarterial chemoembolization (TACE), selective internal radiation therapy (SIRT) and Radiofrequency ablation (RFA) had been performed prior to resection in 31, 6 and 10 cases, respectively. Patients´ characteristics are described in Table 1.

**Table 1:** Patients` characteristics

Age (median; range)	63 (16–92)
Male/female (*n*; %)	1 800 (56.7)/1 377(43.3)
BMI (median; range)	25.9 (15.1–54.9)
ASA (*n*; %)	
I	70 (2.2)
II	1 604 (50.5)
III	1 443 (45.4)
IV	60 (1.9)
Cirrhosis (*n*; %)	194 (6.1)
Child A	188 (6.9)
Child B	6 (0.2)
Arterial hypertension (*n*; %)	1 614 (50.8)
Diabetes mellitus (*n*; %)	546 (17.2)
COPD/asthma (*n*; %)	248 (7.8)
Coronary heart disease (*n*; %)	305 (9.6)
Heart failure/cardiomyopathy (*n*; %)	98 (3.1)
Peripheral vascular disease (*n*; %)	67 (2.1)
Atrial fibrillation (*n*; %)	219 (6.9)

*ASA* American Society of Anesthesiologists risk classification, *BMI* Body-Mass-Index, *COPD* Chronic obstructive pulmonary disease

Hepatic resection was performed mainly for malignant liver disease (*n* = 2 705; 85.1%) including 1 054 (33.2%) resections for primary hepatic or biliary cancer. The most common indications were colorectal liver metastases (CRLM; *n* = 1 265; 39.8%), followed by hepatocellular carcinoma (HCC; *n* = 402; 12.7%), and intrahepatic cholangiocarcinoma (iCCA; *n* = 327; 10.3%; Table 2). In a few cases, especially in benign diseases, there was more than one tumor entity.

**Table 2:** Indications for liver resection

Indication	*n* (%)
Benign	472 (14.9)
Hemangioma	40 (1.3)
Focal nodular hyperplasia	72 (2.3)
Adenoma	50 (1.6)
Echinococcosis	36 (1.1)
Polycystic liver disease/Cysts	148 (4.7)
Other	126 (4.0)
Malignant	2 705 (85.1)
Colorectal metastases	1 265 (39.8)
Hepatocellular carcinoma	402 (12.7)
Intrahepatic cholangiocarcinoma	327 (10.3)
Perihilar cholangiocarcinoma	200 (6.3)
Neuroendocrine metastases	96 (3.0)
Non-colorectal, non-neuroendocrine metastases	288 (9.1)
Gallbladder cancer	81 (2.5)
Primary liver sarcomas	27 (0.8)
Combined hepatocellular, cholangiocellular carcinoma	17 (0.5)
Other	2 (0.1)

Major hepatectomy (defined as resection of ≥ 3 segments) was performed in 1 345 (42.3%) cases. Extrahepatic procedures were performed in 516 (16.2%) cases. Hepaticojejunostomy (biliodigestive anastomosis = BDA) was required in 374 (11.8%) and vascular reconstruction in 301 (9.5%) cases. Simultaneous resection of colorectal primary tumors was conducted in 104 (3.3%). The types of resection and additional procedures are shown in Table 3.

**Table 3:** Operative procedures

Type of resection	*n* (%)
Major resection (≥ 3 segments)	1 345 (42.3)
Right Hemihepatectomy	317 (10.0)
Left Hemihepatectomy	251 (7.9)
Extended right Hemihepatectomy	173 (5.4)
Extended left Hemihepatectomy	117 (3.7)
Mesohepatectomy	81 (2.5)
ALPPS	31 (1.0)
Combination of segments ≥ 3	375 (11.8)
Minor resection	1 832 (57.7)
Left lateral sectionectomy	196 (6.2)
Bisegmentectomy	301 (9.5)
Combination of segments (< 3 segments)	125 (3.9)
Segmentectomy	443 (13.9)
Non-anatomical resection	767 (24.1)
Additional procedures in 1110 patients (34.9%)	
Hepaticojejunostomy	374 (11.8)
Resection of diaphragm	199 (6.3)
Adrenalectomy	43 (1.4)
Simultaneous resection of primary colorectal cancer	104 (3.3)
Simultaneous resection of NET ileum	33 (1.0)
Other	17 (0.5)
Vascular resections/reconstructions	340 (10.7)
Vena cava	90 (2.8)
Portal vein	175 (5.5)
Liver veins	56 (1.8)
Hepatic artery	19 (0.6)

Intraoperative and postoperative blood transfusions were required in 257 (8.1%) and 607 (19.1%) cases, respectively. The need for intraoperative blood transfusion decreased over time (early group 2008–2012: 19.5% vs. late group 2019–2023: 6.1%; *p* < 0.001).

The median duration of the operation was 200 min (range: 29–815 min). The median duration of hospital stay was 10 days (range: 2-169 days) and the median duration of IMC/ICU stay was 1 day (range: 0–75 days).

Pringle-maneuver was used in 718 (22.6%) cases. The use of Pringle-maneuver decreased over time (early/mid group 2008–2018: 36.1% vs. late group 2019–2023: 16.2%; *p* < 0.001). Total vascular occlusion was applied in 47 and in-situ cooling of the liver/ ante situm resection in two cases.

### Morbidity

**Fig. 1:**
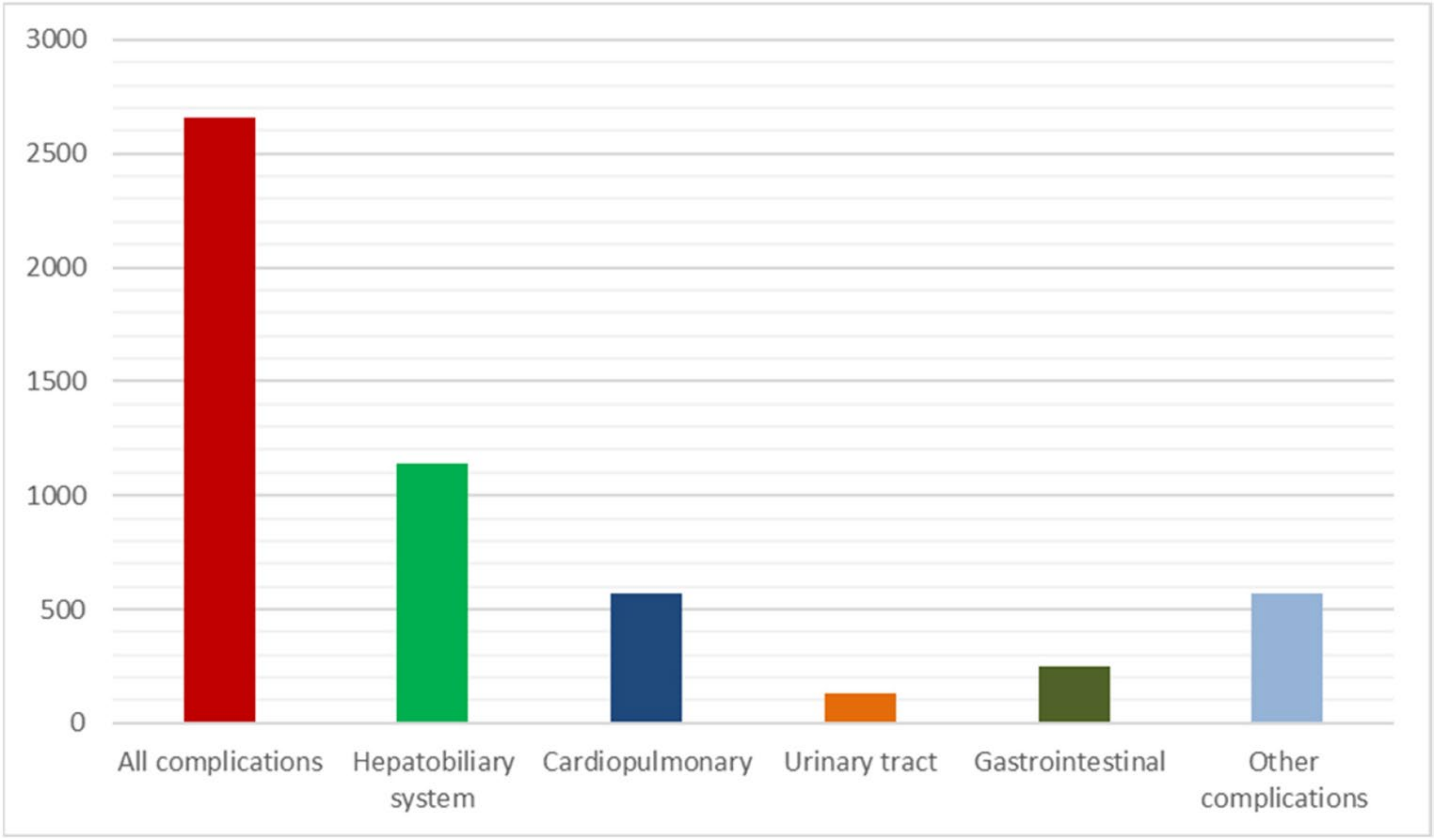
Postoperative complications (n)

In 1 647 cases (51.8%), there was no complication after liver resection. Within 90 days after surgery a total of 2 658 complications (grade I-V according to Clavien-Dindo classification) occurred after 1 530 resections (48.2% of all resections). This included 1 141 complications referring to the hepatobiliary system, 568 cardiopulmonary complications, 133 complications of the urinary tract, 249 gastrointestinal complications and 567 other complications (Fig. 1).

Table 4 provides an overview of all postoperative complications.

**Table 4:** Postoperative complications

Hepatobiliary	
Biloma/bile leakage	490 (15.4)
Perihepatic abscess	144 (4.5)
Hepatic insufficiency/failure	139 (4.4)
Portal vein thrombosis	33 (1.0)
Perihepatic hematoma	46 (1.4)
Ascites/perihepatic fluid collection	143 (4.5)
Cholangitis	86 (2.7)
Lymphatic fistula	37 (1.2)
Pulmonary/cardiovascular	
Symptomatic pleural effusion	197 (6.2)
Pneumonia	132 (4.2)
Pulmonary embolism	45 (1.4)
Respiratory insufficiency	41 (1.3)
Pleural empyema	16 (0.5)
Arrhythmia	58 (1.8)
Thrombosis	20 (0.6)
Urinary tract	
Urinary tract infection	78 (2.5)
Renal failure	55 (1.7)
Gastrointestinal	
Ileus	14 (0.4)
Bowel obstruction	106 (3.3)
Gastrointestinal bleeding	28 (0.9)
General	
Wound infection	263 (8.3)
Wound dehiscence	54 (1.7)
Perioperative bleeding	96 (3.0)
Sepsis	38 (1.2)

**Fig. 2:**
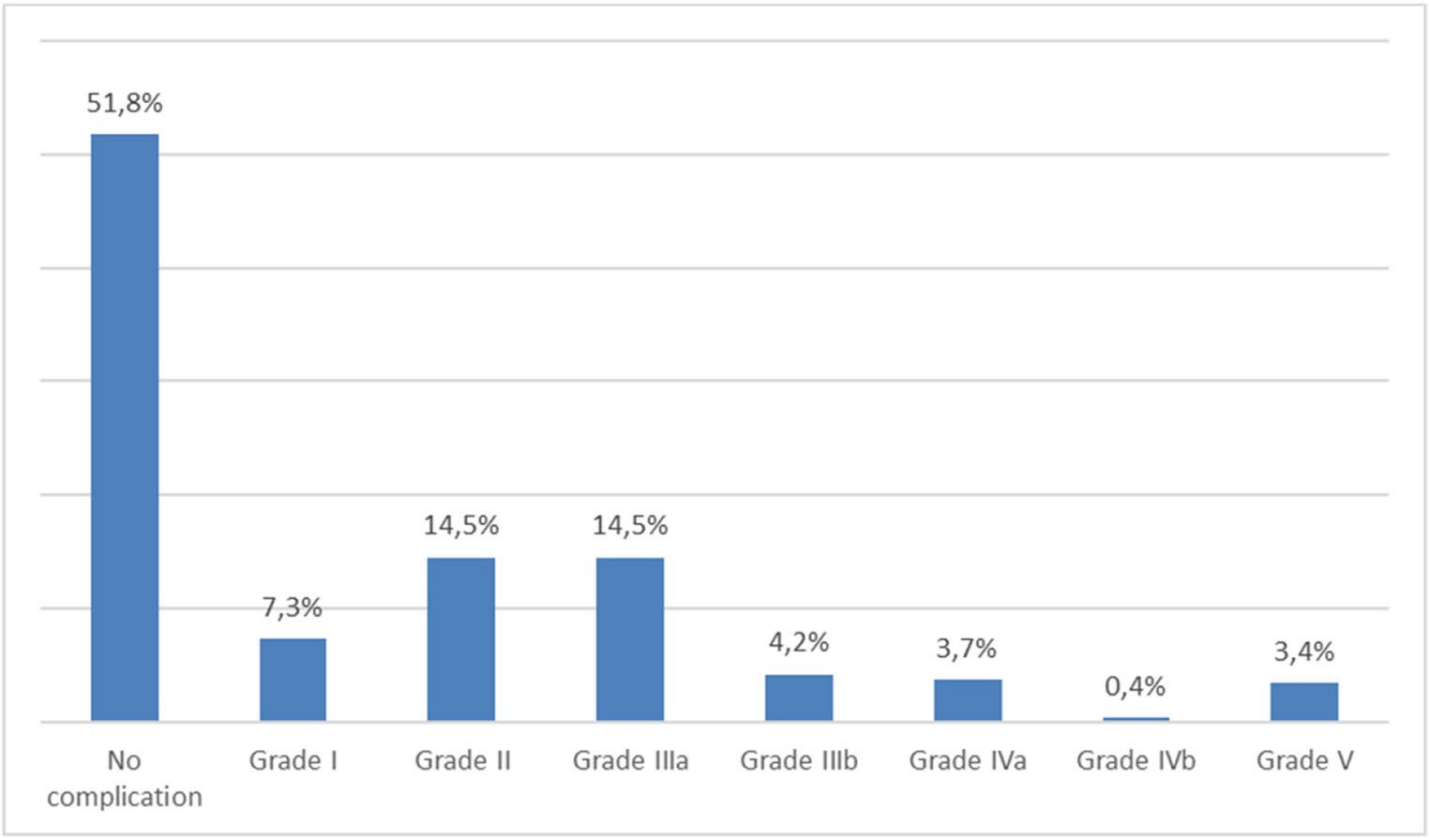
Highest grade of complication (Clavien-Dindo classification)

In 835 (26.3%) cases, patients had one or more complications graded ≥IIIa (Fig. 2).

Major hepatectomy, additional procedures, vascular reconstruction and need for hepaticojejunostomy, as well as age, duration of operation and intraoperative blood transfusion were associated with a higher complication rate in univariate analysis (*p* < 0.001; Table 5).

**Table 5:** Univariate analysis and binary logistic regression of risk factors for morbidity and mortality

	Univariate analysis			Multivariate analysis		
		*p*-value		OR	95% CI	*p*-value
Mortality						
Age (years)	median: 70 vs. 62	< 0.001		0.95	0.94, 0.97	< 0.001
Major hepatectomy	7.0 vs. 1.0%	< 0.001				
Vascular reconstruction	12.3 vs. 2.6%	< 0.001				
Hepaticojejunostomy	15.0 vs. 2.0%	< 0.001		0.2	0.10, 0.38	< 0.001
Duration of operation (min)	median: 363 vs. 196	< 0.001		0.3	0.99; 1.00	0.001
Intraoperative blood transfusion	9.7 vs. 1.7%	< 0.001		0.9	0.18, 0.5	< 0.001
Morbidity						
Age (years)	median: 64 vs. 62	< 0.001		0.98	0.97, 0.99	< 0.001
Major hepatectomy	37.4 vs. 18.2%	< 0.001				
Vascular reconstruction	47.8 vs. 24.1%	< 0.001				
Hepaticojejunostomy	55.9 vs. 22.4%	< 0.001		0.61	0.44, 0.85	0.003
Duration of operation (min)	median: 264 vs. 181	< 0.001		0.99	0.99, 1.00	< 0.001
Intraoperative blood transfusion	46.3 vs. 20.1%	< 0.001		0.56	0.44, 0.71	< 0.001

*OR* Odds Ratio, *CI* Confidence interval

The rate of complications graded ≥ IIIa was 5.5% (19/345) after minimal invasive liver resection, 26% (50/194) in patients with liver cirrhosis and 16.3% (145/892) for parenchyma sparing resections.

In multivariate analysis, age (*p* < 0.001), need for a hepaticojejunostomy (*p* = 0.003), need for intraoperative blood transfusion (*p* < 0.001) and duration of surgery (*p* < 0.001) were independent risk factors for morbidity (Table 5).

Bile leakage was the most frequent complication with 490 (15.4%) cases, followed by wound infection 263 (8.3%), symptomatic pleural effusion 197 (6.2%), perihepatic abscesses and hematoma/hemorrhages in 144 (4.5%) and 142 (4.4%), respectively. Bile leakage was classified as grade A in 169 (5.3%), grade B in 258 (8.1%), and grade C in 63 (2.0%) cases. Grade C bile leaks were due to insufficiency of a BDA (*n* = 11) or because of major leakage at the cutting surface (*n* = 38). Another 14 bile leaks requiring re-operation had developed secondary after operative revision for abscess/bleeding/hematoma.

Hepatic insufficiency (ISGLS criteria) was observed in 139 (4.4%) cases. Of these 56 (1.8%) were classified as grade A, 26 as grade B and 57 (1.8%) as grade C. Using the definition from Mullen et al. [14] posthepatectomy liver failure was present in 50 and using the “50–50 criteria” in 57 (1.8%) cases, respectively.

In cases of parenchyma sparing resection the rate of hepatic insufficiencies (ISGLS criteria) was significantly lower (1.1% vs. 6.2%; *p* < 0.001) than in other hepatectomies. In cases of severe liver failure (grade C), liver failure was associated with small for size syndrome in 10 cases, with vascular complications in 15 cases and with sepsis in 32 cases.

### Mortality

Perioperative 30- and 90-day mortality rates were 2.6% (82) and 3.5% (111), respectively. The lowest mortality rate in malignant indications was observed for colorectal liver metastases, with a perioperative mortality of 1.3% (16/1 265) followed by HCC, with a perioperative mortality of 3.5% (14/402). The perioperative mortality rate in patients with liver cirrhosis was 3.6% (7/194), whereas the mortality rate after minimal-invasive liver resection was 0.6% (2/345). Mortality after parenchyma sparing resections was 1.1% (10/892). Major hepatectomy was associated with a perioperative mortality rate of 7.0% (94/1 345), whereas perioperative mortality for minor hepatectomy was 1.0% (18/1 832; *p* < 0.001). Mortality rate for cases with BDA was 15.0% vs. 2.0% in cases without BDA (*p* < 0.001).

The rates of BDA and vascular reconstruction were 23.5% (vs. 3.2; *p* < 0.001) and 18.1% (vs. 3.2; *p* < 0.001) in group of major hepatectomy.

In the multivariate analysis age (*p* < 0.001), need for hepaticojejunostomy (*p* < 0.001), duration of operation (*p* = 0.001) and need for intraoperative blood transfusion (*p* < 0.001) were independent risk factors for mortality (Table 5). Operative revision was associated with an increased mortality in multivariate analysis (*p* < 0.001), whereas interventional treatment alone was no risk factor for mortality (mortality rate 0.7%; *p* = 0.172).

In the two different time periods from 2008 to 2018 and the last five years from 2019 to 2023, morbidity (complication Grad ≥ III) and mortality were 27.3% versus 24.3% (*p* = 0.091) and 3.7% versus 3.2% (0 = 0.091). In these two periods the need for BDA and vascular resection/reconstruction was 11.3 versus 12.8 (*p* = 0.216) and 9.4% versus 9.8% (*p* = 0.650), respectively. The share of resections of perihilar cholangiocarcinoma increased from 5.4% to 8.2%. While the need for intraoperative blood transfusion significantly decreased from 18.4% to 6.2% (*p* < 0.001), both the share of minimally invasive resections and of parenchymal sparing resections increased from 9% to 14.9% (*p* < 0.001) and from 31.1% to 38.9% (*p* < 0.001), respectively.

In total, 835 cases (26.3%) had major complications grade ≥IIIa. Of these, 727 (87%) cases had major complications grade IIIa-IVb, but could be discharged from hospital (rescue group). Grade V complications occurred in 108 cases (13%) and patients died during hospital stay (failure to rescue group). Age (*p* = 0.003), bilioenteric anastomosis (*p* < 0.001) and need for intraoperative blood transfusion (*p* < 0.001) were independently associated with failure to rescue in multivariate analysis.

### Management of complications

In 735/3 177 (23.1%) a total of 1 158 reinterventions were performed. This included 256 reoperations, 61 s operative revisions, 109 endoscopic procedures, and 732 percutaneous, mainly radiologic guided interventions.

One or more radiologic interventions were performed in 492 patients (15.5%). The most frequent intervention was placement of percutaneous abdominal drainages (PAD; *n* = 429). Indications for PAD were biloma/bile leakage in 223 (7.0%), perihepatic abscesses in 121 (3.8%), hematoma in 31, seroma/ascites in 45 and pancreatic or duodenal fistula in 9 cases (0.3%). Percutaneous transhepatic bile duct drainage (PTBD) was performed in 58 patients, with 19 patients needing a second PTBD. Indications were bile leakage at the cut surface in 23, insufficiency of hepaticojejunostomy in 26, and bile duct stenosis in 9 cases. In total, 51 angiographic interventions were performed, including embolization/coiling (*n* = 31) and stent application (*n* = 20).

The application of pleural drainage or single puncture (*n* = 202) due to symptomatic pleural effusion or empyema was necessary in 178 patients.

Endoscopic interventions were performed in 94 patients. This included 67 ERCP or repeated ERCP (in 62 patients) for bile leakage or bile duct stenosis in 60 and 7 cases, respectively. Furthermore, 13 colonoscopies and 29 gastroscopies were performed in 32 patients because of gastrointestinal bleeding. The median time between initial surgery and first radiologic or endoscopic intervention for bile leakage was 7 days (range: 4–15).

Operative revision was required in 256 (8.1%) patients at a median of 6 days (range: 0–75) after the initial surgery. Reasons for relaparotomy included bleeding and evacuation of hematoma in 68 (2.1%), wound dehiscence in 42, bile leakage in 38, insufficiency of hepaticojejunostomy in 11, portal vein thrombosis in 10, and perihepatic abscesses in 12 cases. 75 re-operations were performed for various indications. A second operative revision was necessary in 61 (23.8%) of these patients.

The median time between initial surgery and first re-operation was 4 days (range: 0–31) for bleeding, 11 days (range: 1–29) for wound dehiscence, 3 days (range: 1–15) for bile leakage, 2 days (range: 1–68) for evacuation of hematoma, 9 days (range: 2–23) for insufficiency of hepaticojejunostomy, 2 days (range: 0–15) for portal vein thrombosis and 8 days (range: 3–47) for perihepatic abscesses.

Operative revision was associated with an increased mortality rate (26.6% vs. 1.5%, *p* < 0.001). There was no significant difference in mortality regarding the time of operative revision (*p* = 0.394).

Thrombosis of portal vein (*n* = 33) was managed with operative thrombectomy (*n* = 10) and, if necessary, re-anastomoses of portal vein at a median of 2 days after initial surgery (range 0–8 days). In these cases, hemodynamic instability and liver failure were the main causes for surgical intervention. In the remaining 23 cases, portal vein thrombosis was managed by radiologic stenting (*n* = 5) or interventional thrombectomy (*n* = 1) plus heparinization or by heparinization only (*n* = 17) after a median of 8 days after initial surgery. In these 23 cases portal vein thrombosis had been partially with a washed around thrombus. Stenosis/thrombosis of hepatic artery (*n* = 2), stenosis/partial thrombosis of Vena cava (*n* = 2) or of a major hepatic vein (*n* = 1) was managed by radiologic recanalization and stenting of the vessel. Half of the patients having surgical revision of portal vein thrombosis and 23% (6/23) with radiologic/conservative management of portal vein thrombosis died in the perioperative period.

Management of liver failure grad B and C included supplementation of albumin, coagulation factors as well as hemodialysis or respiratory ventilation if necessary. In cases of vascular occlusion as cause of hepatic failure radiologic or surgical interventions at the involved vessel were performed.

## Discussion

Over the past decades, hepatic resections have evolved from high-risk surgery in the 1970s to a surgical routine in the ensuing decades [1, 15]. This was followed by a progressive expansion of indications. In the present study, analyzing 3 177 consecutive hepatic resections in a high-volume center, we could demonstrate that despite continuous progress in surgical technique, liver resection is associated with low but still considerable perioperative morbidity and mortality rates. Furthermore, our study could provide a detailed overview about the spectrum of complications after liver resection and the different management strategies, especially including interventional strategies.

In our series, the 90-day mortality of 3.5% was about one third higher than the 30-day-mortality (2.6%). Although this difference is smaller than in other studies which show an almost doubling of mortality in the mentioned period, our findings confirm the limited value of using in-hospital-mortality or 30-day mortality only as definition endpoints [16, 17].

As expected in our analysis we could clearly outline a significant difference in perioperative morbidity and mortality depending on the extent of resection and the underlying disease. The lowest perioperative mortality for resection of malignant tumors was observed in the subgroup of colorectal liver metastases (1.3%), probably due to the increasing use of parenchymal-sparing instead of major resections in our center and a sometimes more restrictive attitude towards extensive resections, in particular in view of other treatment options such as local ablative treatments and, most recently, progress in targeted and immunotherapy [18–20]. This change in operative management is reflected best by the significantly lower rate of PHLF of only 1.1% in parenchyma sparing resections compared to 6.2% in other hepatectomies. Perioperative morbidity and mortality decreased over time, but did not reach statistical significance.

Multivariate analysis identified the patient´s age, the need for hepaticojejunostomy, the duration of the procedure and the intraoperative blood transfusion requirement as independent risk factors for both, postoperative morbidity and mortality. In addition, in univariate analysis major hepatectomy and the need for vascular resection/reconstruction were also associated with increased morbidity and mortality. Our results are completely in line with reports from other high-volume centers who found the need for intraoperative blood transfusion as well as the extent of hepatectomy i.e. the number of resected segments as predictors of postoperative mortality [2–6]. However, these reports are derived from highly specialized single-centers only which bears the risk of selection bias and thus, in contrast to population-based data, not reflecting the entire spectrum of liver resection.

An analysis of outcome data after liver resection in Germany from 2019 revealed a high in-hospital-mortality rate of 5.8% after liver resection [6]. Comparing very high- and low-volume centers the in-hospital mortality rates were 4.6 and 7.5%, respectively, which demonstrates the need for centralization of these complex procedures in Germany.

A population-based Swedish study with a total of 4 460 liver resections, enrolling all liver resections performed in Sweden over a 10-years-period report a 90 day-mortality of 3.1% [21]. More than 80% of resections were performed in university hospitals/high volume centers. The data reveal a significant difference in postoperative mortality between the 7 university and the 33 non-university hospitals with a 30- and 90-day mortality of 1.6% and 2.8% compared to 3.8% and 6.6%, respectively. This difference may be easily explained by an effect of centralization of hepatic resections in specialized centers in Sweden. At first glance, our data with a perioperative mortality rate of 3.5% seem to be a little worse than those of the Swedish University hospitals but further analysis reveals that there is a bias based on underlying diseases and extent of resection. In the Swedish series there was only 10.3% of biliary cancers (iCC, perihilar cholangiocarcinoma, gallbladder carcinoma), while in ours with 19.1% it was almost double. This certainly may explain the somewhat worse results in our series since surgery for bile duct cancer is associated with very high mortality rates, reaching or even exceeding 10% [22–24]. Further, in the Swedish series the percentage of CRLMs (58.8%) was noticeably higher and the percentage of extended resections (7.7%) lower than in the presented study (39.8% CRLMs only, and 9.1% extended resections). Although data are difficult to compare our results seem to support the Swedish findings that patients benefit if liver resections are performed in specialized centers.

The overall morbidity rate in the presented study was 48.2%, meaning that almost every other patient developed one or more complications within 90 days after hepatic surgery (a total of 2 658 complications after 1 530 resections). Our meticulous and detailed analysis of postoperative morbidity is important because despite increasing experience in hepatic surgery there is still a wide range of reported postoperative complications from 12% to 69% [2, 3, 5, 15, 25]. This wide variation certainly is multifactorial and may be explained by different classifications, but also as a result of different surgical aspects such as indication to surgery and patient selection. Rössler et al. reported an incidence of 31% overall complications including a 5% rate of post-hepatectomy liver failure after 5 202 living-donor hepatectomy in 12 centers all around the world [26]. In this study, living-donor hepatectomy was chosen to be a surgical reference of comparison as it is, of course, the highest standard and eventually the most ideal setting for hepatobiliary surgery associated with the best potential outcome. Its comparison to hepatobiliary surgical oncology, however, is in our opinion of limited utility. While living-donor hepatectomy is performed in healthy persons with healthy livers only, hepatectomy usually is performed for malignant disease in patients with comorbidities and in livers with parenchymal damage (steatosis, cirrhosis or after pretreatment with chemotherapy), which adds up in a comparison of non-comparable patients and procedures.

The incidence of bile leakage is reported to range between 4 and 19% [9, 15, 27]. In our study the incidence of bile leakage was 15.4%, which is higher than in reports from other high-volume centers. This could be explained by the larger proportion of patients with biliary tumors and the higher rate of biliary reconstructions, which both are significant risk factors for postoperative bile leaks [28]. Successful management of bile leakage/bilioma by one or repeated non-operative interventions was possible in 258 of our cases. Re-operation was performed for an otherwise not manageable bile leakage only or, rarely, for high volume leaks in the early postoperative period (day 1–3). Grade C bile leakage occurred in 63 of our patients and was associated with a mortality rate of 29.7% due to severe septic constellation and multiorgan failure in our cohort. However, surgical treatment was the last resort and only possible treatment option in these patients because of the severity of the bile leak.

Post-hepatectomy liver failure is another severe complication of liver resection. The incidence of posthepatectomy liver failure has been reported to be between 1.2% and 32% [8, 29]. This high variability in the literature is mainly due to the different definitions of posthepatectomy liver failure. In our study, hepatic insufficiency or failure occurred in 4.4% of cases (ISGLS criteria). Of these patients, 1.8% were classified as grade C and required treatment on intensive care unit. Most of these cases were associated with septic constellation or were observed after vascular thrombosis, mainly of portal vein thrombosis. Comparing different definitions, we found that in our study the incidence of severe liver failure (requiring a change of patients` clinical management) was almost identically, no matter which definition was applied (ISGLS definition (1.8%), “50–50 criteria” (1.8%) and definition of Mullen et al. (1.6%) [8, 13, 14]. This indicates the validity of these three definitions.

In our series, nearly half of the complications were Clavien-Dindo Grade I and II not requiring any treatment or could be managed conservatively. Half of the other half of complications were classified as grade ≥IIIa. Our data with a need for one or more radiologic interventions in 15.5% of cases is considerably higher than in a report from the USA where in a total of 11 243 patients over a 3-years period radiologic intervention was necessary in 6% only [30]. But the series from the US included only 30-day morbidity and a smaller rate of biliary reconstruction (only 6.5%). In our study, operative revision was necessary in 8.1% of cases and associated with a significantly increased mortality rate of 26.6%, most-likely due to dense adhesions and followed by a secondary inflammatory reaction [31]. Although there is a huge bias towards impaired outcome after surgical revision, re-operation with general anesthesia should be reserved for cases unsuccessfully managed by intervention or when inevitable due to substantial hemorrhage, or early vascular thrombosis associated with liver failure or hemodynamic instability.

Over the study period (early/mid period 2008–2018 and late period 2019–2023) there was a slight but statistically not significant trend towards a lower morbidity and mortality, although there was a higher percentage of resections of perihilar cholangiocarcinoma and a greater need for hepaticojejunostomies and vascular reconstructions in the second period (2019–2023). Our findings are similar to reports from other high-volume centers where morbidity and mortality decreased over time while complexity of resections increased [32]. On the other hand, in the second period both the rate of parenchyma sparing resections for CRLM as well as minimally invasive resections were higher. Notably, there were slight but constant changes in surgical technique over time i.e. a trend to a lesser use of Pringle maneuver and vascular occlusion, as well as in preoperative imaging, in particular a more precise planning of complex resection in case of multifocal tumors by a more frequent use of 3-D-reconstructions or even 3-D-prints. This has been paralleled by a continuous improvement of intra- and postoperative anesthesiologic management as well as steady progress in interventional management of complications. It is difficult to attribute the trend to a lower morbidity and mortality to a specific factor but certainly, an ongoing analysis of complications with the aim to mitigate incidence and severity of intra- and postoperative problems has contributed to this slight improvement.

Several limitations should be considered when interpreting our data. Although data were collected prospectively, the study has a retrospective nature and as such is probably encountering several selection biases and therefore cannot determine optimal management of complications. However, as age, need for hepaticojejunostomy and duration of operation (reflecting the operations complexity) were independent risk factors for morbidity and mortality, an operation within this constellation should be considered carefully. Data of high-volume centers also bear the risk of tending to more complex resections, thus not reflecting the entire spectrum of liver resection. In addition, it cannot be completely ruled out that some minor complications, in particular Clavien-Dindo grade I might have been overseen thus resulting in a maybe too low number of total complications.

## Conclusion

Despite ongoing improvements in hepatobiliary surgery, postoperative morbidity after liver resection remains substantial. In this large single-center series, advanced age, biliary reconstruction, prolonged operative time, and intraoperative blood transfusion were consistently associated with impaired postoperative outcomes, reflecting increasing procedural complexity and patient-related risk. An operation within this constellation should be considered carefully.

The majority of postoperative complications, particularly biliary complications, could be managed successfully by radiologic and endoscopic approaches. In contrast, the need for surgical revision identified a subgroup of patients with markedly unfavorable outcomes, most likely reflecting the severity of the underlying complication rather than the intervention itself.

These findings highlight the importance of careful perioperative risk stratification, early detection of complications, and a stepwise management strategy prioritizing minimally invasive approaches whenever feasible, while preserving surgical procedures as an essential option in selected life-threatening situations.

## Data Availability

No datasets were generated or analysed during the current study.

## References

[1] Ong GB, Lee NW (1975) Hepatic resection. Br J Surg 62:421–430 167899 10.1002/bjs.1800620602

[2] Jarnagin WR, Gonen M, Fong Y, DeMatteo RP, Ben-Porat L, Little S et al (2002) Improvement in perioperative outcome after hepatic resection: analysis of 1803 consecutive cases over the past decade. Ann Surg 236:397–406 12368667 10.1097/01.SLA.0000029003.66466.B3 PMC1422593

[3] Dimick JB, Pronovost PJ, Cowan JA Jr, Lipsett PA (2003) Postoperative complication rates after hepatic resection in Maryland hospitals. Arch Surg 138:41–46 12511147

[4] Asiyanbola B, Chang D, Gleisner AL, Nathan H, Choti MA, Schulick RD et al (2008) Operative mortality after hepatic resection: are literature-based rates broadly applicable? J Gastrointest Surg 12:842–851 18266046 10.1007/s11605-008-0494-y

[5] Breitenstein S, DeOliveira ML, Raptis DA, Slankamenac K, Kambakamba P, Nerl J et al (2010) Novel and simple preoperative score predicting complicationas after liver resection in noncirrhotic patients. Ann Surg 252:726–734 21037427 10.1097/SLA.0b013e3181fb8c1a

[6] Filmann N, Walter D, Schadde E, Bruns C, Keck T, Lang H et al (2019) Mortality after liver surgery in Germany. Br J Surg 106:1523–1529 31339558 10.1002/bjs.11236

[7] Bagante F, Ruzzenente A, Beal EW, Campagnaro T, Merath K, Conci S et al (2019) Complications after liver surgery: a benchmark analysis. HPB (Oxford) 21:1139–1149 30718185 10.1016/j.hpb.2018.12.013

[8] Rahbari NN, Garden OJ, Padbury R, Brooke-Smith M, Crawford M, Adam R et al (2011) Posthepatectomy liver failure: a definition and grading by the International Study Group of Liver Surgery (ISGLS). Surgery 149:713–724 21236455 10.1016/j.surg.2010.10.001

[9] Koch M, Garden OJ, Padbury R, Rahbari NN, Adam R, Capussotti L et al (2011) Bile leakage after hepatobiliary and pancreatic surgery: a definition and grading of severity by the International Study Group of Liver Surgery. Surgery 149:680–688 21316725 10.1016/j.surg.2010.12.002

[10] Dindo D, Demartines N, Clavien PA (2004) Classification of surgical complications: a new proposal with evaluation in a cohort of 6336 patients and results of a survey. Ann Surg 240:205–213 15273542 10.1097/01.sla.0000133083.54934.ae PMC1360123

[11] Huber T, Huettl F, Tripke V, Baumgart J, Lang H (2021) Experiences with three-dimensional printing in complex liver surgery. Ann Surg 273:e26–e27 33074891 10.1097/SLA.0000000000004348

[12] Stockmann M, Lock JF, Malinowski M, Niehues SM, Seehofer D, Neuhaus P (2010) The LiMAx test: a new liver function test for predicting postoperative outcome in liver surgery. HPB (Oxford) 12:139–146 20495659 10.1111/j.1477-2574.2009.00151.x PMC2826673

[13] Balzan S, Belghiti J, Farges O, Ogata S, Sauvanet A, Delefosse D et al (2005) The 50–50 criteria on postoperative day 5: an accurate predictor of liver failure and death after hepatectomy. Ann Surg 242:824–829 16327492 10.1097/01.sla.0000189131.90876.9e PMC1409891

[14] Mullen JT, Ribero D, Reddy SK, Donadon M, Zorzi D, Gautam S et al (2007) Hepatic insufficiency and mortality in 1,059 noncirrhotic patients undergoing major hepatectomy. J Am Coll Surg 204:854–862 17481498 10.1016/j.jamcollsurg.2006.12.032

[15] Poon RT, Fan ST, Lo CM, Liu CL, Lam CM, Yuen WK et al (2004) Improving perioperative outcome expands the role of hepatectomy in management of benign and malignant hepatobiliary diseases: analysis of 1222 consecutive patients from a prospective database. Ann Surg 240:698–708 discussion 708 – 10 15383797 10.1097/01.sla.0000141195.66155.0c PMC1356471

[16] Mayo SC, Shore AD, Nathan H, Edil BH, Hirose K, Anders RA et al (2011) Refining the definition of perioperative mortality following hepatectomy using death within 90 days as the standard criterion. HPB (Oxford) 13:473–482 21689231 10.1111/j.1477-2574.2011.00326.x PMC3133714

[17] Farges O, Goutte N, Bendersky N, Falissard B, ACHBT-French Hepatectomy Study Group (2012) Incidence and risks of liver resection: an all-inclusive French nationwide study. Ann Surg 256:697–704 discussion 704-5 23095612 10.1097/SLA.0b013e31827241d5

[18] Angelsen JH, Horn A, Sorbye H, Eide GE, Løes IM et al (2017) Population-based study on resection rates and survival in patients with colorectal liver metastasis in Norway. Br J Surg 104:580–589 28181674 10.1002/bjs.10457

[19] Torzilli G, McCormack L, Pawlik T (2020) Parenchyma-sparing liver resections. Int J Surg 82S:192–197 32335245 10.1016/j.ijsu.2020.04.047

[20] Lordan JT, Roberts JK, Hodson J, Isaac J, Muiesan P, Mirza DF et al (2017) Case-controlled study comparing peri-operative and cancer-related outcomes after major hepatectomy and parenchymal sparing hepatectomy for metastatic colorectal cancer. HPB (Oxford) 19:688–694 28495437 10.1016/j.hpb.2017.04.007

[21] Gilg S, Sparrelid E, Isaksson B, Lundell L, Nowak G, Strömberg C (2017) Mortality-related risk factors and long-term survival after 4460 liver resections in Sweden- a population-based study. Langenbecks Arch Surg 402:105–113 27695941 10.1007/s00423-016-1512-2 PMC5309267

[22] Ratti F, Marino R, Olthof PB, Pratschke J, Erdmann JI, Neumann UP et al (2024) Predicting futility of upfront surgery in perihilar cholangiocarcinoma: machine learning analytics model to optimize treatment allocation. Hepatology 79:341–354 37530544 10.1097/HEP.0000000000000554

[23] Lang H, Baumgart J, Heinrich S, Huber T, Heuft LK et al (2021) Liver resection for intrahepatic cholangiocarcinoma-single-center experience with 286 patients undergoing surgical exploration over a thirteen year period. J Clin Med 10:3559 34441855 10.3390/jcm10163559 PMC8396970

[24] Olthof PB, Bouwense SAW, Bednarsch J, Dewulf M, Kazemier G, Maithel S et al (2025) Failure to rescue after resection of perhilar cholangiocarcinoma in an international multicenter cohort. Ann Surg Oncol 32:1762–1768 39404989 10.1245/s10434-024-16293-7 PMC11811460

[25] Spolverato G, Ejaz A, Yuhree K, Weiss M, Wolfgang CL, Hirose K et al (2014) Readmission incidence and associated factors after a hepatic resection at a major hepato-pancreatico-biliary academic centre. HPB (Oxford 16:972–978 24712690 10.1111/hpb.12262 PMC4487747

[26] Rössler F, Sapisochin G, Song G, Lin YH, Simpson MA, Hasegawa K et al (2016) Defining benchmarks for major liver surgery: a multicenter analysis of 5202 living liver donors. Ann Surg 264:492–500 27433909 10.1097/SLA.0000000000001849

[27] Yamashita Y, Hamamatsu T, Rikimaru T, Tanaka S, Shirabe K, Shimada M et al (2001) Bile leakage after hepatic resection. Ann Surg 233:45–50 11141224 10.1097/00000658-200101000-00008 PMC1421165

[28] Martin AN, Narayanan S, Turrentine FE, Bauer TW, Adams RB, Stukenborg GJ et al (2018) Clinical factors and postoperative impact of bile leak after liver resection. J Gastrointest Surg 22:661–667 29247421 10.1007/s11605-017-3650-4 PMC5871550

[29] Lafaro K, Buettner S, Maqsood H, Wagner D, Bagante F, Spolverato G et al (2015) Defining post hepatectomy liver insufficiency: where do we stand? J Gastrointest Surg 19:2079–2092 26063080 10.1007/s11605-015-2872-6

[30] Kolarich AR, Solomon AJ, Weiss MJ, Philosophe B, Weiss CR, Hong K (2021) Risk factors for complications requiring interventional radiological treatment after hepatectomy. J Gastrointest Surg 25:1184–1192 32462493 10.1007/s11605-020-04609-3

[31] Pace RF, Blenkharn JI, Edwards WJ, Orloff M, Blumgart LH, Benjamin IS (1989) Intraabdominal sepsis after hepatic resection. Ann Surg 209:302–306 2493775 10.1097/00000658-198903000-00009 PMC1493942

[32] Zimmitti G, Roses RE, Andreou A, Shindoh J, Curley SA, Aloia TA et al (2013) Greater complexity of liver surgery is not associated with an increased incidence of liver-related complications except for bile leakage: An experience with 2628 consecutive resections. J Gastrointest Surg 17:57–65 22956403 10.1007/s11605-012-2000-9 PMC3855461

